# Supplementary material for: Aesthetic evaluation and the perceived properties of Chinese characters
Source: PLoS One. 2025 Jan 31;20(1):e0318353. doi: 10.1371/journal.pone.0318353 (PMC11785276; doi:10.1371/journal.pone.0318353)
Supplement: S1 File — Procedure of pilot experiment. (DOCX) [file pone.0318353.s001.docx]

## **Pilot experiment**

### **Participants**

Six participants were recruited from Kyushu University and were paid 1,000 yen for their participation. Three were native Japanese speakers and three were native Chinese speakers. The mean age of the participants was 24.7 (SD = 2.16) years old (3 females). All the participants provided written informed consent before participating in the experiment. All the participants had normal or corrected-to-normal vision.

### **Apparatus and Materials**

The experiment was conducted in a sound-attenuating chamber. Stimuli were presented on a 21.9-inch (46 × 29 cm) LCD monitor (EIZO CG223W). The stimulus presentation and data collection were controlled using a personal computer (EPSON Endeavor MR4300E). Stimuli were displayed on the Chrome browser software using jsPsych (Leeuw et al., 2023) and were presented in black (luminance: 0.12 cd/m^2^) at the center of the screen with a height of 200 pixels. The instructional text was black (0.12 cd/m^2^), the choice framelines were dark gray (9.10 cd/m2), and the background was light gray (42.6 cd/m^2^). A set of 52 Chinese ideographs, randomly selected from Japanese common Kanji list, were presented in Regular script (楷体_GB2312).

### **Procedure**

In this experiment, the participants were asked to observe the screen from a distance of 57 cm with their heads fixed using a chin rest. The participants first rated whether the stimulus was beautiful using a 3-point Likert scale (1 = not beautiful, 2 = neutral, and 3 = beautiful). Participants were then asked to rate the emotional valence of the stimuli (1 = negative, 2 = neutral, and 3 = positive). All stimuli were presented in a randomized order. The total number of trials was 104 (52 Chinese characters × 2 tasks), which were divided into four blocks (26 trials × 4 tasks). Five practice trials were conducted prior to each task.

### **Analysis**

Data from six participants were analyzed. To avoid the influence of emotional valence in the formal experiments, we selected stimuli with neutral emotional valence (1.5 < mean value of emotional valence < 2.5). Furthermore, stimuli in which aesthetic and emotional valence ratings differed from those of native Japanese and Chinese speakers were excluded (absolute differences < 1). Fig 1 shows the set of 18 Chinese characters selected for Experiments 1 and 2.
