# Supplementary material for: Aesthetic evaluation and the perceived properties of Chinese characters
Source: PLoS One. 2025 Jan 31;20(1):e0318353. doi: 10.1371/journal.pone.0318353 (PMC11785276; doi:10.1371/journal.pone.0318353)
Supplement: S2 File — (DOCX) [file pone.0318353.s002.docx]

**S2 File. Results of the GLMM applied to the aesthetic evaluation in Experiment 1 for each font.**

Semi-cursive

## Linear mixed model fit by REML. t-tests use Satterthwaite's method [
## lmerModLmerTest]
## Formula: beauty ~ 1 + symmetry + complexity + prototype + (1 | id)
## Data: dat_f
##
## REML criterion at convergence: 1325.7
##
## Scaled residuals:
## Min 1Q Median 3Q Max
## -3.3669 -0.6921 0.0707 0.5707 3.0375
##
## Random effects:
## Groups Name Variance Std.Dev.
## id (Intercept) 0.4116 0.6416
## Residual 0.4251 0.6520
## Number of obs: 612, groups: id, 34
##
## Fixed effects:
## Estimate Std. Error df t value Pr(>|t|)
## (Intercept) 5.224e-01 1.151e-01 3.480e+01 4.538 6.49e-05 ***
## symmetry -1.516e-04 3.387e-02 5.919e+02 -0.004 0.99643
## complexity 1.293e-02 3.353e-02 5.957e+02 0.386 0.69989
## prototype 1.036e-01 3.799e-02 6.039e+02 2.727 0.00658 **
## ---
## Signif. codes: 0 '***' 0.001 '**' 0.01 '*' 0.05 '.' 0.1 ' ' 1
##
## Correlation of Fixed Effects:
## (Intr) symmtr cmplxt
## symmetry -0.013
## complexity -0.072 0.386
## prototype 0.149 -0.137 0.150

Regular

## Linear mixed model fit by REML. t-tests use Satterthwaite's method [
## lmerModLmerTest]
## Formula: beauty ~ 1 + symmetry + complexity + prototype + (1 | id)
## Data: dat_f
##
## REML criterion at convergence: 1331.1
##
## Scaled residuals:
## Min 1Q Median 3Q Max
## -3.2167 -0.5858 0.1346 0.6156 3.3816
##
## Random effects:
## Groups Name Variance Std.Dev.
## id (Intercept) 0.2525 0.5025
## Residual 0.4400 0.6633
## Number of obs: 612, groups: id, 34
##
## Fixed effects:
## Estimate Std. Error df t value Pr(>|t|)
## (Intercept) 0.63739 0.09032 32.88252 7.057 4.56e-08 ***
## symmetry 0.01225 0.03227 592.85439 0.380 0.7044
## complexity 0.06408 0.03302 592.63791 1.941 0.0527 .
## prototype 0.02067 0.03293 599.92878 0.628 0.5305
## ---
## Signif. codes: 0 '***' 0.001 '**' 0.01 '*' 0.05 '.' 0.1 ' ' 1
##
## Correlation of Fixed Effects:
## (Intr) symmtr cmplxt
## symmetry 0.017
## complexity 0.012 0.338
## prototype -0.033 -0.006 0.254

Gothic

## Linear mixed model fit by REML. t-tests use Satterthwaite's method [
## lmerModLmerTest]
## Formula: beauty ~ 1 + symmetry + complexity + prototype + (1 | id)
## Data: dat_f
##
## REML criterion at convergence: 1234.3
##
## Scaled residuals:
## Min 1Q Median 3Q Max
## -3.0391 -0.6276 -0.1160 0.7163 3.7973
##
## Random effects:
## Groups Name Variance Std.Dev.
## id (Intercept) 0.3062 0.5533
## Residual 0.3684 0.6069
## Number of obs: 612, groups: id, 34
##
## Fixed effects:
## Estimate Std. Error df t value Pr(>|t|)
## (Intercept) -0.401020 0.098249 33.233906 -4.082 0.000264 ***
## symmetry -0.003283 0.027196 581.187754 -0.121 0.903972
## complexity 0.104744 0.030595 590.151693 3.424 0.000661 ***
## prototype 0.088293 0.032700 595.445984 2.700 0.007130 **
## ---
## Signif. codes: 0 '***' 0.001 '**' 0.01 '*' 0.05 '.' 0.1 ' ' 1
##
## Correlation of Fixed Effects:
## (Intr) symmtr cmplxt
## symmetry -0.007
## complexity 0.015 0.365
## prototype -0.062 -0.048 0.121

Maru Gothic

## Linear mixed model fit by REML. t-tests use Satterthwaite's method [
## lmerModLmerTest]
## Formula: beauty ~ 1 + symmetry + complexity + prototype + (1 | id)
## Data: dat_f
##
## REML criterion at convergence: 1222.1
##
## Scaled residuals:
## Min 1Q Median 3Q Max
## -2.2591 -0.4636 -0.0938 0.4519 3.6392
##
## Random effects:
## Groups Name Variance Std.Dev.
## id (Intercept) 0.2354 0.4852
## Residual 0.3657 0.6048
## Number of obs: 612, groups: id, 34
##
## Fixed effects:
## Estimate Std. Error df t value Pr(>|t|)
## (Intercept) -7.171e-01 8.705e-02 3.333e+01 -8.239 1.51e-09 ***
## symmetry -6.068e-04 2.667e-02 5.846e+02 -0.023 0.98186
## complexity 9.809e-02 3.184e-02 5.947e+02 3.081 0.00216 **
## prototype 4.870e-02 3.120e-02 6.056e+02 1.561 0.11908
## ---
## Signif. codes: 0 '***' 0.001 '**' 0.01 '*' 0.05 '.' 0.1 ' ' 1
##
## Correlation of Fixed Effects:
## (Intr) symmtr cmplxt
## symmetry -0.008
## complexity 0.074 0.331
## prototype 0.042 -0.096 0.158

Mincho

## Linear mixed model fit by REML. t-tests use Satterthwaite's method [
## lmerModLmerTest]
## Formula: beauty ~ 1 + symmetry + complexity + prototype + (1 | id)
## Data: dat_f
##
## REML criterion at convergence: 1429.9
##
## Scaled residuals:
## Min 1Q Median 3Q Max
## -2.63187 -0.56200 -0.08285 0.70882 2.68223
##
## Random effects:
## Groups Name Variance Std.Dev.
## id (Intercept) 0.3370 0.5806
## Residual 0.5143 0.7171
## Number of obs: 612, groups: id, 34
##
## Fixed effects:
## Estimate Std. Error df t value Pr(>|t|)
## (Intercept) -1.263e-04 1.044e-01 3.376e+01 -0.001 0.999042
## symmetry -2.029e-02 3.230e-02 5.850e+02 -0.628 0.530103
## complexity 1.213e-01 3.478e-02 5.903e+02 3.487 0.000525 ***
## prototype 1.128e-01 3.943e-02 5.945e+02 2.861 0.004368 **
## ---
## Signif. codes: 0 '***' 0.001 '**' 0.01 '*' 0.05 '.' 0.1 ' ' 1
##
## Correlation of Fixed Effects:
## (Intr) symmtr cmplxt
## symmetry -0.012
## complexity -0.011 0.369
## prototype -0.112 0.034 0.182
