# Supplementary material for: Aesthetic evaluation and the perceived properties of Chinese characters
Source: PLoS One. 2025 Jan 31;20(1):e0318353. doi: 10.1371/journal.pone.0318353 (PMC11785276; doi:10.1371/journal.pone.0318353)
Supplement: S3 File — (DOCX) [file pone.0318353.s003.docx]

**S3 File. Results of the GLMM applied to the aesthetic evaluation in Experiment 2 for each script.**

Semi-cursive

## Linear mixed model fit by REML. t-tests use Satterthwaite's method [
## lmerModLmerTest]
## Formula: beauty ~ 1 + symmetry + complexity + prototype + experience +
## (1 | randomID)
## Data: dat_f
##
## REML criterion at convergence: 3393.1
##
## Scaled residuals:
## Min 1Q Median 3Q Max
## -3.8009 -0.7273 0.0580 0.6333 3.1529
##
## Random effects:
## Groups Name Variance Std.Dev.
## randomID (Intercept) 0.2576 0.5075
## Residual 0.5374 0.7331
## Number of obs: 1440, groups: randomID, 80
##
## Fixed effects:
## Estimate Std. Error df t value Pr(>|t|)
## (Intercept) 4.111e-01 6.003e-02 7.814e+01 6.847 1.52e-09 ***
## symmetry 3.108e-02 2.304e-02 1.415e+03 1.349 0.178
## complexity 2.383e-02 2.540e-02 1.430e+03 0.938 0.348
## prototype 2.419e-01 2.416e-02 1.417e+03 10.012 < 2e-16 ***
## experience 5.632e-02 6.002e-02 7.805e+01 0.938 0.351
## ---
## Signif. codes: 0 '***' 0.001 '**' 0.01 '*' 0.05 '.' 0.1 ' ' 1
##
## Correlation of Fixed Effects:
## (Intr) symmtr cmplxt prttyp
## symmetry 0.013
## complexity -0.017 0.304
## prototype -0.054 -0.079 0.317
## experience -0.001 -0.048 -0.020 0.007

Regular

## Linear mixed model fit by REML. t-tests use Satterthwaite's method [
## lmerModLmerTest]
## Formula: beauty ~ 1 + symmetry + complexity + prototype + experience +
## (1 | randomID)
## Data: dat_f
##
## REML criterion at convergence: 3038.8
##
## Scaled residuals:
## Min 1Q Median 3Q Max
## -3.4699 -0.5693 0.0723 0.5008 4.1002
##
## Random effects:
## Groups Name Variance Std.Dev.
## randomID (Intercept) 0.2549 0.5049
## Residual 0.4150 0.6442
## Number of obs: 1440, groups: randomID, 80
##
## Fixed effects:
## Estimate Std. Error df t value Pr(>|t|)
## (Intercept) 6.560e-01 6.545e-02 1.157e+02 10.023 < 2e-16 ***
## symmetry 7.110e-03 1.921e-02 1.417e+03 0.370 0.711
## complexity 4.394e-02 2.525e-02 1.432e+03 1.740 0.082 .
## prototype 1.300e-01 3.084e-02 1.433e+03 4.215 2.65e-05 ***
## experience 1.867e-02 5.907e-02 7.831e+01 0.316 0.753
## ---
## Signif. codes: 0 '***' 0.001 '**' 0.01 '*' 0.05 '.' 0.1 ' ' 1
##
## Correlation of Fixed Effects:
## (Intr) symmtr cmplxt prttyp
## symmetry 0.077
## complexity 0.096 0.312
## prototype -0.407 -0.110 0.136
## experience -0.015 -0.057 -0.026 0.029

Cursive

## Linear mixed model fit by REML. t-tests use Satterthwaite's method [
## lmerModLmerTest]
## Formula: beauty ~ 1 + symmetry + complexity + prototype + experience +
## (1 | randomID)
## Data: dat_f
##
## REML criterion at convergence: 3319.3
##
## Scaled residuals:
## Min 1Q Median 3Q Max
## -3.2907 -0.6422 -0.1064 0.6537 3.7644
##
## Random effects:
## Groups Name Variance Std.Dev.
## randomID (Intercept) 0.3575 0.5979
## Residual 0.5008 0.7076
## Number of obs: 1440, groups: randomID, 80
##
## Fixed effects:
## Estimate Std. Error df t value Pr(>|t|)
## (Intercept) -0.01277 0.07087 83.66788 -0.180 0.857455
## symmetry -0.05115 0.02672 1419.62371 -1.914 0.055789 .
## complexity -0.08247 0.02475 1426.83738 -3.333 0.000882 ***
## prototype 0.25193 0.02912 1406.36774 8.651 < 2e-16 ***
## experience 0.06974 0.06949 77.45161 1.004 0.318658
## ---
## Signif. codes: 0 '***' 0.001 '**' 0.01 '*' 0.05 '.' 0.1 ' ' 1
##
## Correlation of Fixed Effects:
## (Intr) symmtr cmplxt prttyp
## symmetry 0.021
## complexity 0.038 0.132
## prototype 0.191 -0.131 0.378
## experience 0.002 -0.046 0.004 0.021

Clerical

## Linear mixed model fit by REML. t-tests use Satterthwaite's method [
## lmerModLmerTest]
## Formula: beauty ~ 1 + symmetry + complexity + prototype + experience +
## (1 | randomID)
## Data: dat_f
##
## REML criterion at convergence: 3073.4
##
## Scaled residuals:
## Min 1Q Median 3Q Max
## -2.6521 -0.7259 -0.0456 0.6186 3.7506
##
## Random effects:
## Groups Name Variance Std.Dev.
## randomID (Intercept) 0.1844 0.4294
## Residual 0.4325 0.6577
## Number of obs: 1440, groups: randomID, 80
##
## Fixed effects:
## Estimate Std. Error df t value Pr(>|t|)
## (Intercept) -0.45916 0.05128 79.04523 -8.954 1.19e-13 ***
## symmetry 0.08825 0.01855 1396.82282 4.757 2.17e-06 ***
## complexity 0.03316 0.02305 1434.47960 1.439 0.15046
## prototype 0.08010 0.02515 1426.51226 3.184 0.00148 **
## experience 0.09721 0.05113 78.12632 1.901 0.06095 .
## ---
## Signif. codes: 0 '***' 0.001 '**' 0.01 '*' 0.05 '.' 0.1 ' ' 1
##
## Correlation of Fixed Effects:
## (Intr) symmtr cmplxt prttyp
## symmetry 0.013
## complexity 0.060 0.165
## prototype -0.041 -0.187 0.407
## experience -0.004 -0.041 -0.011 0.040

Seal

## Linear mixed model fit by REML. t-tests use Satterthwaite's method [
## lmerModLmerTest]
## Formula: beauty ~ 1 + symmetry + complexity + prototype + experience +
## (1 | randomID)
## Data: dat_f
##
## REML criterion at convergence: 2530.4
##
## Scaled residuals:
## Min 1Q Median 3Q Max
## -2.8901 -0.6003 -0.0978 0.4910 5.1381
##
## Random effects:
## Groups Name Variance Std.Dev.
## randomID (Intercept) 0.2899 0.5384
## Residual 0.2837 0.5326
## Number of obs: 1440, groups: randomID, 80
##
## Fixed effects:
## Estimate Std. Error df t value Pr(>|t|)
## (Intercept) -0.55337 0.06386 88.40774 -8.666 1.93e-13 ***
## symmetry -0.01748 0.01504 1383.91016 -1.162 0.245
## complexity 0.03628 0.01756 1403.89764 2.066 0.039 *
## prototype 0.09133 0.02323 1394.37280 3.932 8.84e-05 ***
## experience 0.08323 0.06184 77.90935 1.346 0.182
## ---
## Signif. codes: 0 '***' 0.001 '**' 0.01 '*' 0.05 '.' 0.1 ' ' 1
##
## Correlation of Fixed Effects:
## (Intr) symmtr cmplxt prttyp
## symmetry -0.035
## complexity -0.070 0.213
## prototype 0.214 0.020 0.258
## experience 0.003 -0.024 -0.013 0.002
